# Monitoring Public Perception of Health Risks in Brazil and Italy: Cross-Cultural Research on the Risk Perception of Choking in Children

**DOI:** 10.3390/children8070541

**Published:** 2021-06-24

**Authors:** Alexander Hochdorn, Alexia Oliveira, Giulia Lorenzoni, Andrea Francavilla, Solidea Baldas, Paola Berchialla, Alessandra Oliveira, Vicente Paulo Alves, Dario Gregori, Danila Azzolina

**Affiliations:** 1 Department of Social and Work Psychology, University of Brasília, Brasília 72220-275, Brazil; alexander.hochdorn@gmail.com; 2 School of Medicine and Healthcare Sciences, Catholic University of Brasília, Brasília 72220-275, Brazil; alexia_v.o@hotmail.com (A.O.); a.oliveira53@gmail.com (A.O.); vicerap@gmail.com (V.P.A.); 3 Unit of Biostatistics, Epidemiology and Public Health, Department of Cardiac Thoracic Vascular Sciences and Public Health, University of Padua, 35131 Padua, Italy; giulia.lorenzoni@unipd.it (G.L.); andrea.francavilla@studenti.unipd.it (A.F.); danila.azzolina@ubep.unipd.it (D.A.); 4 Prochild ONLUS, 34129 Trieste, Italy; solidea.baldas@prochild.eu; 5 Department of Clinical and Biological Sciences, University of Torino, 10124 Torino, Italy; paola.berchialla@unito.it; 6 Department of Medical Science, University of Ferrara, 44121 Ferrara, Italy

**Keywords:** (accidental) foreign body injuries/object injuries, hazard-choking, cross-cultural study

## Abstract

One of the most relevant public health issues among pediatric injuries concerns foreign body (FB) aspiration. The risk perception of choking hazards (CH) and risk perception, in general, are complex multifactorial problems that play a significant role in defining protective behavior. Risk prevention policies should take this aspect into account. A lack of scientific knowledge of FB injury risk perception may be evidenced in Brazil and other newly developed countries. This study aims to characterize the differences and peculiarities in risk perception of CH between Italian and Brazilian populations. The risk perception among adults in Italy and Brazil between September and October 2017 was investigated in a survey. A Multiple Correspondence Analysis was carried out to identify the latent components characterizing the risk perception in Italian and Brazilian population samples. The most relevant dimension characterizing risk perception is the “Professional–educational status and the related perception of Risk” (13% of factorial inertia). The Italians identify batteries and magnets as the most dangerous choking risks (20% of responses). On the other hand, Brazilian people, mainly manual laborers (22%) with secondary or primary education (94%), perceive coins as the most dangerous items (30% of responses, *p* < 0.001). Socio-economic issues characterize the subjective risk perception of Italian and Brazilian survey respondents. In this framework, data-driven prevention strategies could be helpful to tailor intervention strategies to the cultural context to which they are addressed.

## 1. Introduction

Risk perception, which defines how people think and feel about the risks they face, represents a cognitive issue and plays a significant role in protective behavior [1,2]. Risk awareness is a multidimensional and complex aspect influenced by psychological and socio-cultural features [3,4,5,6]. The World Health Organization (WHO) specifies that reality’s knowledge and experience deeply influence hazard perception, which is eventually associated with cultural aspects [7,8].

Given the complexity of risk perception, prevention policies should be tailored to the specific social framework [9]. Conversely, worldwide intervention policies are usually managed with general and non-specific guidelines [7]. In many circumstances, prevention activities are based on education [10]. This is the case of preventing childhood injuries, where policies are still in progress in the so-called Newly Industrialized Countries [11].

One of the most relevant public health issues among pediatric injuries concerns foreign body (FB) aspiration. In most cases, FBs are expelled spontaneously [12], but a not negligible proportion of FBs get stuck in the aero-digestive tract, leading to severe complications [13].

Referring to the Brazilian context, different studies have been conducted to evaluate the prevalence of unintentional injuries and FB aspiration injuries in preschool children [14]. Some aimed to investigate the prevalence of FB ingestion in Brazilian settings [15]. In contrast, others (i.e., the WHO study) investigated the FB injury prevalence in Brazil and additional newly developed countries, focusing on a global perspective [16].

However, a lack of scientific knowledge and prevention policies concerning FB injuries may be evidenced, mainly in Brazil and in newly developed countries [17]. These considerations justify the need to start up a pilot survey in Brazil to compare the results with a similar survey conducted in Italy, representing a developed country.

The immediate identification of a lack of interventions is crucial for the efficient implementation of culturally tailored prevention strategies. Handy tools offering an immediate picture of the intervention policy optimality have been poorly implemented. Recently, data-mining instruments have been proposed in the literature to provide a multidimensional comparison among countries in the field of epidemiology of FB injuries [18]. Previous research aimed to analyze the characteristics of FB injuries in Bosnia and Herzegovina, a newly industrialized country, by comparing cases from the other European countries via Multiple Correspondence Analysis (MCA) [18].

This paper aims to broaden the application of the existing tools to monitor the risk perception with potential impact on public health policies. The main idea is that once a specific country has been tested for prevention policies, other nations can be compared with it, and whatever differences are evidenced, if any, can be interpreted as an early signal of lack of implementations. Once the shortcomings in risk prevention policies have been identified, the intervention to be implemented should be tailored to the socio-economic context to which they refer [8,18].

Italy is at the cutting edge of a prevention strategy. In this direction, recent efforts were made on regulations, disseminating activities (safe food), and targeted initiatives to schools. For example, the Italian Choking Prevention project (CHOP) [19] was established to identify the most effective educational strategy for preventing FB injuries. The prevention policy’s actions should also consider the parent’s risk perception, which is an essential and culture-related element [7].

This work aims to characterize the main aspects of risk perception of choking hazard (CH) through an MCA analysis of Italy and Brazil as performed in similar research contexts for cross-cultural comparability purposes [18]. Italy was considered a benchmark because the country has implemented consolidated prevention strategies in the CH prevention area, while Brazil is a newly developed country where little is known about the practices adopted to prevent choking hazard events. Brazil still does not have enough tools to reduce the rate of aspiration of foreign bodies. The latest FB injury data from the Ministry of Health date back to 2012 [20]; the data revealed that 756 children up to 14 years old died from suffocation. The injuries caused by ingestion or aspiration of foreign bodies remain a serious public health problem in the pediatric population [21].

## 2. Materials and Methods

### 2.1. Study Design and Setting

#### Data Collection Instrument

The questionnaire has been developed in the context of the Susy Safe project [22] and has been used in other research settings as well [23]. It is administered to collect data on the risk perception of CH among adults having no formal health degree. The data collection tool includes the principal sociodemographic information: gender, age, employment type, and educational attainment.

The questionnaire includes ten images of jewelry, popcorn, batteries, hotdogs, toys, stationery, candies, coins, nuts, and seeds. These are the same items of the questionnaire previously administered in Italy [23]; they seem to represent most of the CH findings, particularly in a child aged 0–3 years [17].

Participants were asked to look at the images and answer by classifying a maximum of two items (per age group) that they considered to be the highest CH for children aged (A) less than 1-year-old; (B) 1–2 years old; and (C) 3–6 years old. According to the literature, the age classification has been defined considering that the risk of injury is highest in children between 1 and 2 years of age [24]. Additional details about the data collection tool are reported elsewhere [23].

All subjects gave their informed consent for inclusion before they participated in the study. The study was conducted following the Declaration of Helsinki and approved by the local ethics committee (“Commissao Nacional de Ètica em Pasquisa” project identification code = 17988619.7.0000.5553).

No compensation was provided for the survey participants in both the Italian and Brazilian samples.

### 2.2. Adults without Health Education Survey

*Italian sample*. The questionnaire was administered to 742 subjects not having a specific academic qualification in any health field between 2014 and 2016. The inclusion criteria were age over 18 years and the ability to read and speak Italian. The questionnaire was self-administered with a pencil and paper. Subjects were enrolled during multiple national events between 2014 and 2016. These public events aimed to sensitize adult supervisors on the issue of food choking injuries in children [23]. Only Italian respondents were selected to perform the analysis. Other details about questionnaire administration in the Italian sample can be found elsewhere [23].

*Brazilian sample.* The questionnaire was administered to 172 subjects without a specific degree in healthcare sciences in 2017. The inclusion criteria were age over 18 years and the ability to understand and speak Portuguese. The questionnaire was not self-administrated since not all respondents could read and write in their native language.

The questionnaire was translated into Portuguese with the help of a committee [25]. In this case, two translators worked together to produce a consensus.

All subjects have been recruited in the University Hospital of Ceilândia, a densely inhabited satellite town of Brazil’s Federal Capital Brasília.

### 2.3. Statistical Analysis

#### 2.3.1. Multiple Correspondence Analysis

An approach based on Multiple Correspondence Analysis (MCA) was chosen to characterize respondents. MCA is a statistical method used to create multivariate contingency tables, and thus it does not require the definition of a dependent variable. MCA aims to summarize associations between variables in large, potentially complex datasets [26].

Orthogonal factorial axes (latent dimensions) were extracted in descending order, explaining the highest variability of the data matrix.

The results were summarized in a two-dimensional subspace reporting the respective coordinates of individuals (respondents) and categories (variable modalities).

For this study, individual and variable MCA summaries were reported:

For individuals, the relative coordinates were graphically reported in a biplot to identify similar groups according to their characteristics recognized by latent dimensions. A comparison between Italy and Brazil was performed in terms of median factor loadings (coordinates) regarding selected axes.

For variable modality, the results were summarized considering the relative coordinate and contribution of each variable’s modality to the axis’s definition to identify the main characteristics contributing to the latent dimension.

The standardized coordinates, presented as test values, were used to define specific characterization on each side of the latent dimensions. If the test value’s absolute value for one particular variable modality is superior to 2, then the specified coordinate is significantly different from zero [27].

MCA was performed using the R packages FactomineR [28] and Factoextra [29].

#### 2.3.2. Descriptive Statistics

Descriptive statistics were carried out according to the children’s country of origin (Italian vs. Brazil). Categorical data were reported as relative and absolute frequencies, continuous data as median and I and III quartiles. Wilcoxon rank sum tests were performed for continuous variables and the Pearson’s Chi-square test, or Fisher’s exact test, whatever appropriate, were used for categorical variables. Analyses were performed using R 3.5.2 [30].

## 3. Results

Overall, 742 Italian and 172 Brazilian questionnaires were administered (Table 1). In Italy, 79% of the respondents were female compared to 92% in the Brazilian survey (*p* < 0.001).

The differences in occupational compositions of the two samples are significant (*p* < 0.001); the majority of Brazilian respondents were manual laborers (35%) or were self-employed (22%). In Italy, instead, the greater part of the sample comprises office workers (35%) and managers (27%). In the Brazilian sample, 33% of subjects have at least three children, while, in the Italian sample, this proportion is smaller and equal to 12% (*p* < 0.001). According to the educational level, 6% of Brazilian respondents hold post-degree educational attainment compared to 41% of Italian respondents (*p* < 0.001).

The Italian sample perceived the batteries and toys as the most hazardous items for their children aged less than one year. Brazilians perceived coins and jewelry instead as more harmful. The second lead choice for Italians was “coins and candies”; while Brazilians chose “coins and seed” (Table 1).

Considering the risk perception for 1- and 2-year-old children, the Italians consider the most dangerous FB items to be batteries and toys. Coins and toys were the first choices for Brazilians. The second-choice distribution is consistent with the responses provided for the children aged less than 1 year (Table 1).

For 3-year-old and older children, the Italians perceive toys and batteries as the most dangerous objects. Brazilians instead think the most dangerous ones are toys and coins. The second choice is candies and coins for Italians, and stationery and coins for Brazilian respondents (Table 1).

A higher proportion of Italian respondents (31%) experienced choking accidents among their children compared to Brazilians (22%), *p* = 0.024 (Table 1). Among Italians, injury accidents were mainly due to food ingestion, 81% vs. 26% among Brazilians (*p* < 0.001), and a smaller proportion of them involved the risk of death (12% vs. 32%, *p* < 0.001) (Table 1).

Most Italian adults (86%), having at least one child, experienced the risk of choking at least once in comparison with the Brazilian sample (*p* = 0.024) (Table S1). Among them, a considerable proportion of adults has more than four children (24%), *p* < 0.001 (Table S1, Supplementary Materials).

The 9% of the adults experiencing a choking hazard for their child perceived batteries as the second hazardous item compared to the 3% in the remaining sample (*p* = 0.027) (Table S1, Supplementary Materials).

Finally, comparing Italian and Brazilian countries, the frequency of adults in managerial or teaching positions reporting FB injury episodes in their children is similar (Table S2, Supplementary Materials).

### MCA Results

The first two latent dimensions explain 20% of the overall variability. The first latent dimension explains most of the overall inertia (13%). The most important contributors to the first dimension are the professional status of respondents (self-employment working status) and educational attainment (primary education Level) (Table 2).

The contributors to the second factor (Table 2) are more related to choking risk perception in infants aged less than 1 year.

The information provided by the latent factors could be synthesized by considering the most contributing variables on the latent dimensions [18] (Table 2). For example, the first factor has been named “Professional–educational status and perception of Risk”, for the reason that the socio-economic features contribute the most to a variable’s modalities. The “Factors affecting Risk perception in children < 1 year” (Table 2) are instead the most contributing variable modalities on the second latent factor.

A further interpretation of latent axes may be performed with standardized coordinates (test values) to specify respondents’ peculiarities according to their locations on selected axes.

Considering the first dimension, on the negative side of the factor (negative test values), it is possible to identify two groups of respondents characterized as Italian males (median loading −0.24) with a high education level, in managerial employment without children. This group of respondents identifies batteries as a more hazardous object for children in all age classes (Table 2).

On the positive side of these factors (positive test values), Brazilian respondents are reported, where (median loading 0.84) their primary education was classified as own worker or manual worker, and the number of children was greater than four. Brazilian respondents identify coins as the most dangerous objects (Table 2).

According to the second latent dimension, it is possible to distinguish, on the positive side, the subjects identifying a potential FB injury hazard in toys and popcorn. On the negative side instead, hot dogs and batteries are identified as most hazardous.

**Figure 1:**
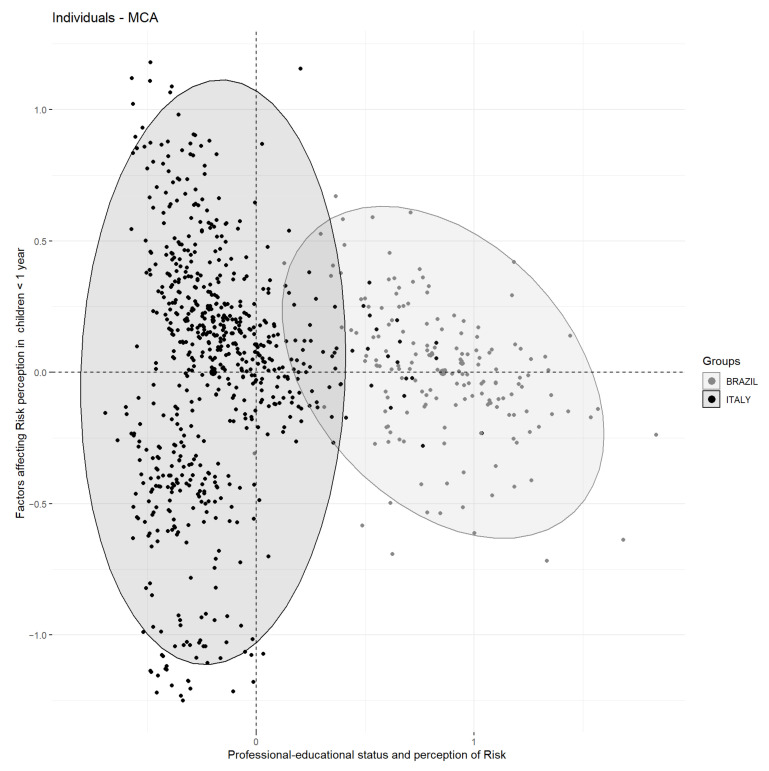
MCA individual biplot for dimensions 1 (*x*-axis) and 2 (*y*-axis), according to the children’s country of origin (Italy vs. Brazil).

The differences in geographical and cultural belonging are mainly evident in the first latent factor (the most important in terms of explained variance) (Figure 1).

## 4. Discussion

This work shows that some socio-economic peculiarities in the Brazilian context are connected to a different definition of risk perception of choking injuries compared to the Italian setting.

Some studies conducted on injury prevention in Brazil showed that some social groups and individuals are at higher risk for unintentional injuries [31]. The identified situations at risk are heterogeneous, and they may include aspects related to education, salary, access to health services, unemployment, or the absence of a family network. The latter was shown to be fundamental, particularly in families with a larger number of children [32].

Considering the peculiarities of FB injuries risk perception, the results showed that Italians perceived both batteries and magnets as more harmful for their children. Conversely, the Brazilian respondents identify a higher risk in coins and buttons. Epidemiological surveillance is helpful to define the configurations of choking injuries in children [33]. The literature shows that the majority of FB injuries in children are related to coins and marble ingestion [34], but the most dangerous items are magnets [35] and batteries [36], due to the increased chance of severe complications.

The prevention policies recently implemented in Italy have raised public awareness about the serious complications arising from the ingestion of batteries and magnets [19].

Concerning the results of this work, the Brazilian respondents perceive coins, which are among the main causes of incidents across children [36], as more dangerous. This risk perception probably derives from personal experience rather than from an actual risk awareness, mainly because coins are much more common in daily life for Brazilian respondents than batteries.

In our research, the Italians declare that the FB injury events are mainly due to the ingestion of food items compared to the Brazilian sample who reported a greater number of events due to non-food items. The literature confirmed that, in European countries, most FB injuries are caused by inorganic items [23,37], and food items, in particular nuts and seeds, are the most frequently retrieved from a child’s upper airway tract [34].

Other research has shown that most FB injury events in European countries occur during a meal when the adult is distracted. In newly developed countries, where policies are less focused on the topic, FB injury happens mainly under adult supervision while the child is playing [18]. This highlights a lack of awareness of the problem in the Brazilian sample, which over-represents the non-food items among the objects involved in FB injuries and underestimates the perception of risk associated with ingestion of batteries and magnets.

Research conducted in an emergency ward of a general hospital in Brazil underlined that most FB injuries among children occur in the home environment where many items and situations can establish risk scenarios [38]. In some situations, inadequate child supervision is related to the fact that the adult responsible for the caregiving activities is simultaneously involved in other activities at the same time. Adult presence does not prevent FB injuries; research demonstrated that 4.3% of the children presenting in the emergency room were alone when the incident occurred [38]. This could happen when, even though the adult caregiver is available for supervision, he lacks the understanding of what constitutes a threat to the child. In these situations, the caregiver could inadvertently allow the child to play with dangerous items, such as batteries and magnets.

A considerable part of the Brazilian sample belongs to low-income classes [39,40]. The people originating from underdeveloped urban areas, mostly Black or Brown people, with poor educational levels, have limited access to healthcare and social policies. As pointed out by different authors [39,40], the levels of awareness regarding situations representing a considerable risk for one’s physical and/or psychological well-being are extremely precarious. Indeed, a relevant percentage of all Brazilian mothers, who took part in the survey, were very young at the moment of data collection, sometimes having more than one child.

Inexperience, precarious socio-economic backgrounds and low educational levels are often co-responsible for everyday injuries, which could be avoided in more socially advantaged situations and conditions. Considering a certain problem or phenomenon concerning its socio-cultural background, an intersectional view is indispensable for healthcare, social, and psychological research carried out in countries with a post-colonial past, such as the Americas.

Despite the greater number of Italian respondents, compared to Brazilians, declaring to have experienced FB injuries in their children, a greater percentage of events with a fatal outcome is reported in the Brazilian sample. The literature shows that recall of events can be influenced by actual risk awareness [41]. In this regard we could highlight two aspects. On the one hand is the greater awareness of the problem in Italy, shaped by the recent FB-related prevention policies, which probably facilitated the respondent’s recall of events that involved less serious outcomes.

On the other hand, the Brazilian respondents, less aware of the problem, could be prone to remember only the events involving more severe outcomes.

The literature shows that the majority of FB injuries in children are related to coins and marble ingestion [34], but the most dangerous items are magnets [35] and batteries [36], due to the increased chance of severe complications. The hazard related to these latter items is underestimated, in our data, by the Brazilian respondents.

Mortality due to foreign body injury is in fact a serious problem in Brazil; FB airway obstruction is the leading cause of death from external causes in infants up to 1 year of age. Most of the cases treated were children around the first 3 years of life, with a prevalence of boys, in a ratio of 2:1 [21]. In Italy, instead, the number of children hospitalized for FB injuries [33] has decreased in the last decade, but at the same time, the awareness of the problem has improved [23].

Our data demonstrated that by performing a comparison between Italian and Brazilian countries, the frequency of adults in managerial or teaching positions reporting FB injury episodes in their children is similar between Italy and Brazil. This could illustrate the fact that a demographic–occupational position could be an issue that defines attitudes and perceptions of risk in a similar way in Italy and Brazil. These results can be viewed as a starting point of some preliminary consideration; preventive actions and targeted policies should be necessary for Brazil to increase awareness and education about the real risk concerning FB injuries.

Compared to European countries, Brazil is characterized by higher levels of socio-cultural differences (economic, educational, and ethnic). In this context, the application of prevention policies can be of crucial importance, especially if the attempt is to reach the economically more vulnerable population groups [42,43]. The application of prevention policies, especially if oriented towards awareness and education about the problem, can reach, with focused interventions, even the most disadvantaged segments of the population. However, the policies do not act alone in this direction. Preventive actions would be easier to implement if the educational substrate of the population is homogeneous and if the population is already oriented to inform itself about public health issues [44].

Concerning FB injury prevention, a few simple measures that could be implemented by any population group, disadvantaged or not, would be helpful. For example, the FB injury events in several cases involve objects present in the children’s homes; it can be assumed that modification of the domestic environment could be an important preventive issue, as well as the adoption of safe behaviors and adequate surveillance habits by parents and caregivers [45]. Within this general framework, guidelines and prevention strategies oriented to the child’s developmental stage could be essential. Certainly, a dual intervention including prevention policies but also the promotion of equal access to education and employment can make the difference [46].

Our questionnaire focused on the socio-demographic peculiarities of the analyzed sample. However, culture is a broader concept that defines the customary beliefs, social forms, and material traits of a racial, religious, or social group [47]. Brazil is characterized by a wider heterogeneity of ethnic–religious groups in comparison with Italy. The country is a cultural patchwork; it was officially colonized by the Portuguese and, at the same time, it had distinctive African and indigenous influences [48]. Italians, French, Dutch, Arabs, Japanese, and other ethnic groups moved to Brazil in the course of its history [49]. This cultural heterogeneity impacts risk perception [50]. Furthermore, it is difficult to discuss Brazil without mentioning the social exclusion connected to the cultural differences; many ethnic groups still face severe limitations in accessing education, social integration, labor market, and healthcare facilities [51].

We have highlighted in our research that the occupational status (manual laborer status) with a lower level of education and a higher number of children for Brazilian respondents defines a different perception of risk compared to the Italians who tend to have a more homogeneous cultural connotation.

The literature demonstrates that [52] the general lack of attention during parental caregiving might cause more FB injury cases, especially among disadvantaged populations [53]. Therefore, special attention should be paid not only to improve the overall awareness within the closer family context but also to enhance the professional caregiving experience [54].

Moreover, Brazilian parents are younger than European parents and only a few of them were engaged in caring for one child. A significant proportion of them carry out extra-domestic and physically demanding full-time jobs. In this setting, families may have difficulties in appropriately supervising children; this is a relevant issue because the literature confirmed that a momentary lack of attention is one of the most common causes of FB injury in children [13,37,55,56,57]. In that regard, a relevant target of prevention is related to parental awareness and generally to a collectively shared recognition of a health problems in the pediatric age [58].

However, the literature from newly industrialized countries [17,59] or Eastern European nations [60,61], demonstrates that extensive and comprehensive prevention policies on the topic are still in progress [60].

The injury prevention policies should be tailored to the socio-economic peculiarities of the targeted population [8,18]. The principal contribution of this survey is to underline the socio-cultural differences between Italy (a developed country) and Brazil (a newly developed country). Cultural peculiarities are considered factors influencing the risk perception and, subsequently, the epidemiology of FB injuries in the pediatric age.

Such intersectional and transnational perspectives could help in tailoring the educational interventions to a specific cultural setting [62]. Therefore, this study could be considered one of the first attempts to analyze an epidemiologically relevant problem through an intersectional, post-modern, and intercultural perspective.

### Study Limitations

The first study limitation consists of a possible incomparability between a relatively small national European context, i.e., Italy, and a macro-national Brazilian context. A single state—the Federal District—was considered to resolve this geopolitical inequality. Most educated people and college students are in the Brazilian Federal District (Brasília—17.6%), exactly where most participants are sampled for many psychological studies [63].

From a statistical standpoint, the current survey concerns the asymmetric distribution of the sample sizes. Such a difference is due to the exploratory character of this type of research. However, MCA allows accounting for both small sample sizes [64] and unbalanced data [65].

Our study findings warrant future research to compare risk perception at the population level between different European countries and the Brazilian population.

## 5. Conclusions

Socio-economic factors are important issues concerning subjective risk perception of FB injuries. Different socio-cultural characteristics of Italian and Brazilian survey respondents were evidenced by our data. A data-driven and cross-cultural approach may be helpful or is recommended in designing injury prevention policies. The intervention policies should address the heterogeneity and complexity of risk perception.

## Figures and Tables

**Table 1:** Characteristics of Italian and Brazilian respondents. Continuous data are reported as median (I, III quartiles); categorical data are reported as percentages and absolute frequencies. Wilcoxon rank sum tests were performed for continuous variables and the Pearson’s Chi-square test, or Fisher’s exact test, whatever appropriate, for categorical variables.

Variable	N	Brazil	Italy	Combined	*p*-Value
		(N = 172)	(N = 742)	(N = 914)	
Gender: Female	902	159 (92%)	575 (79%)	734 (81%)	<0.001
Male		13 (8%)	155 (21%)	168 (19%)	
Age	904	32.0 (25.0–39.0)	42.0 (37.0–49.0)	41.0 (34.0–48.0)	<0.001
Job: Housewife	807	19 (11%)	62 (10%)	81 (10%)	<0.001
Unemployed		17 (10%)	31 (5%)	48 (6%)	
Teacher/Office Worker		24 (14%)	221 (35%)	245 (30%)	
Self-employed		60 (35%)	29 (5%)	89 (11%)	
Manager		2 (1%)	172 (27%)	174 (22%)	
Manual laborer		38 (22%)	63 (10%)	101 (13%)	
Retired		2 (1%)	24 (4%)	26 (3%)	
Student		10 (6%)	33 (5%)	43 (5%)	
Number of children: 0	887	1 (1%)	103 (14%)	104 (12%)	<0.001
1–2		105 (61%)	529 (74%)	634 (71%)	
3–4		56 (33%)	83 (12%)	139 (16%)	
>4		10 (6%)	0 (0%)	10 (1%)	
Education: Higher education	895	104 (60%)	371 (51%)	475 (53%)	<0.001
Primary education		58 (34%)	56 (8%)	114 (13%)	
Post-secondary education		10 (6%)	296 (41%)	306 (34%)	
Hazardous items for children < 1 year (first response): Batteries	894	5 (3%)	144 (20%)	149 (17%)	<0.001
Candies		4 (2%)	129 (18%)	133 (15%)	
Coins		51 (30%)	34 (5%)	85 (10%)	
Hotdog		3 (2%)	31 (4%)	34 (4%)	
Jewelry		56 (33%)	135 (19%)	191 (21%)	
Nuts		1 (1%)	9 (1%)	10 (1%)	
Popcorn		18 (10%)	62 (9%)	80 (9%)	
Seeds		15 (9%)	4 (1%)	19 (2%)	
Stationery		4 (2%)	12 (2%)	16 (2%)	
Toys		15 (9%)	162 (22%)	177 (20%)	
Hazardous items for children < 1 year (second response): Batteries	677	1 (5%)	33 (5%)	34 (5%)	0.069
Candies		1 (5%)	126 (19%)	127 (19%)	
Coins		9 (45%)	219 (33%)	228 (34%)	
Hotdog		0 (0%)	11 (2%)	11 (2%)	
Jewelry		0 (0%)	0 (0%)	0 (0%)	
Nuts		0 (0%)	70 (11%)	70 (10%)	
Popcorn		1 (5%)	20 (3%)	21 (3%)	
Seeds		7 (35%)	77 (12%)	84 (12%)	
Stationery		0 (0%)	29 (4%)	29 (4%)	
Toys		1 (5%)	72 (11%)	73 (11%)	
Hazardous items for children aged 1–2 years (first response): Batteries	888	7 (4%)	167 (23%)	174 (20%)	<0.001
Candies		11 (6%)	119 (17%)	130 (15%)	
Coins		54 (31%)	22 (3%)	76 (9%)	
Hotdog		2 (1%)	51 (7%)	53 (6%)	
Jewelry		33 (19%)	68 (9%)	101 (11%)	
Nuts		3 (2%)	9 (1%)	12 (1%)	
Popcorn		11 (6%)	74 (10%)	85 (10%)	
Seeds		4 (2%)	3 (0%)	7 (1%)	
Stationery		10 (6%)	30 (4%)	40 (5%)	
Toys		37 (22%)	173 (24%)	210 (24%)	
Hazardous items for children aged 1–2 years (second response): Batteries	677	2 (10%)	20 (3%)	22 (3%)	<0.001
Candies		2 (10%)	190 (29%)	192 (28%)	
Coins		6 (29%)	193 (29%)	199 (29%)	
Hotdog		0 (0%)	26 (4%)	26 (4%)	
Jewelry		0 (0%)	0 (0%)	0 (0%)	
Nuts		1 (5%)	85 (13%)	86 (13%)	
Popcorn		3 (14%)	13 (2%)	16 (2%)	
Seeds		5 (24%)	27 (4%)	32 (5%)	
Stationery		1 (5%)	34 (5%)	35 (5%)	
Toys		1 (5%)	68 (10%)	69 (10%)	
Hazardous items for children aged 3–6 years (first response): Batteries	886	15 (9%)	167 (23%)	182 (21%)	<0.001
Candies		22 (13%)	119 (17%)	141 (16%)	
Coins		30 (18%)	22 (3%)	52 (6%)	
Hotdog		8 (5%)	51 (7%)	59 (7%)	
Jewelry		12 (7%)	68 (9%)	80 (9%)	
Nuts		4 (2%)	9 (1%)	13 (1%)	
Popcorn		9 (5%)	74 (10%)	83 (9%)	
Seeds		7 (4%)	3 (0%)	10 (1%)	
Stationery		21 (12%)	30 (4%)	51 (6%)	
Toys		42 (25%)	173 (24%)	215 (24%)	
Hazardous items for children aged 3–6 years (second response): Batteries	676	1 (5%)	20 (3%)	21 (3%)	0.01
Candies		2 (10%)	190 (29%)	192 (28%)	
Coins		8 (40%)	193 (29%)	201 (30%)	
Hotdog		1 (5%)	26 (4%)	27 (4%)	
Jewelry		0 (0%)	0 (0%)	0 (0%)	
Nuts		0 (0%)	85 (13%)	85 (13%)	
Popcorn		0 (0%)	13 (2%)	13 (2%)	
Seeds		4 (20%)	27 (4%)	31 (5%)	
Stationery		3 (15%)	34 (5%)	37 (5%)	
Toys		1 (5%)	68 (10%)	69 (10%)	
Have any children in your household ever experienced risk of choking?: No	840	134 (78%)	462 (69%)	596 (71%)	0.024
Yes		38 (22%)	206 (31%)	244 (29%)	
What object caused the accident?: Food	222	10 (26%)	149 (81%)	159 (72%)	<0.001
Non-Food		28 (74%)	35 (19%)	63 (28%)	
Involving risk of death: No	480	26 (68%)	391 (88%)	417 (87%)	0.001
Yes		12 (32%)	51 (12%)	63 (13%)	

**Table 2:** Contribution (percentage) and test values (standardized coordinates) of variables’ levels for each of the two dimensions. In bold, the most important contributors are defined as variables’ levels with a greater percentage of explained data variance. In bold are also reported the greater test values (absolute values) on both positive and negative sides of the axes. The medians of coordinates of factors contributing to each dimension are for “Professional-educational status and perception of Risk” (Dim 1): 0.83 (Brazil) and −0.24 (Italy); for “Factors affecting Risk perception in children <1 year” (Dim 2): 0.01 (Brazil) and 0.07 (Italy).

Categories	Professional−Educational Status and Perception of Risk (Dim 1)		Factors Affecting Risk Perception in Children <1 Year (Dim 2)	
	Contributions	Test Value	Contributions	Test Value
Gender: Female	0.23	2.67	0.23	−2.01
Male	1.03	**−2.67**	1.02	2.01
Job: Housewife	0.06	0.62	0.4	−1.19
Unemployed	0.48	1.7	0.18	−0.78
Teacher/Office Worker	1.64	−3.65	0.03	−0.34
Self-employed	**5.4**	**5.85**	0.08	−0.53
Manager	1.98	**−3.78**	0.28	1.07
Manual laborer	1.36	**2.96**	0.26	−0.98
Retired	0.08	−0.69	0.83	1.66
Student	0.01	−0.22	1.22	2.04
Number of children: 0	1.13	**−2.68**	2.93	3.27
1–3	0.24	−2.17	0.28	−1.79
3–4	2.25	3.87	0.04	−0.39
>4	2.99	**4.13**	0.27	−0.94
Education: Higher education	0	−0.1	0.08	0.75
Primary education	**6.18**	**6.32**	0.16	−0.76
Post-secondary education	2.19	**−4.33**	0.01	−0.26
Hazardous items for children < 1 year (first response): Batteries	1.93	**−3.62**	3.45	−3.65
Candies	0.99	−2.56	2.08	2.8
Coins	3.65	**4.77**	0.15	−0.72
Hotdog	0.09	−0.74	0.01	0.17
Jewelry	1.45	3.22	0.01	0.17
Nuts	0.07	−0.65	0.53	1.31
Popcorn	0.26	1.26	0.15	−0.72
Seeds	3.53	4.51	0.02	0.28
Stationery	0.15	0.93	0.35	1.07
Toys	1.72	−3.48	0.2	0.9
Hazardous items for children < 1 year (second response): Batteries	3.08	**−4.65**	8.39	−5.8
Candies	0.11	−0.85	0.52	1.4
Coins	**9.61**	**7.7**	0	0.04
Hotdog	0.56	−1.83	**16.56**	**−7.53**
Jewelry	1.37	2.95	0.05	0.41
Nuts	0.01	−0.21	2.63	2.93
Popcorn	0.58	−1.9	**10.16**	**6.02**
Seeds	1.57	2.99	0.1	−0.58
Stationery	0.8	2.17	0.12	−0.63
Toys	0.31	−1.51	3.03	3.58
Hazardous items for children aged 1–2 years (first response): Batteries	2.16	**−3.92**	8.55	−5.89
Candies	0	0.09	0.47	1.34
Coins	**5.36**	**5.67**	0.12	0.64
Hotdog	0.3	−1.35	**16.52**	**−7.55**
Jewelry	0.22	1.17	0.11	0.64
Nuts	0	0.04	2.73	2.99
Popcorn	0.7	−2.09	9.75	**5.89**
Seeds	1.77	3.17	0.09	−0.53
Stationery	2.07	3.51	0.13	−0.68
Toys	0.08	−0.77	3.18	**3.68**
Have any children in your household ever experienced risk of choking?: no	0	0.18	0.42	2.16
yes	0	−0.18	1.03	−2.16
What object caused the accident?: Food	0.14	−1.68	0	0.07
Non-Food	0.36	1.68	0	−0.07
Involving risk of death: No	0.02	−0.99	0.01	0.61
Yes	0.15	0.99	0.1	−0.61

## Data Availability

Data will be made available upon motivated request to the authors.

## Supplementary Materials

The following are available online at https://www.mdpi.com/article/10.3390/children8070541/s1, Table S1: Characteristics and item choices for adults having and not having children ever experienced risk of choking, Table S2: Adults having any children in the household ever experienced risk of choking in managerial position/office and position/teachers category in Italy and Brazil.
