# Supplementary material for: Monitoring Public Perception of Health Risks in Brazil and Italy: Cross-Cultural Research on the Risk Perception of Choking in Children
Source: Children (Basel). 2021 Jun 24;8(7):541. doi: 10.3390/children8070541 (PMC8307887; doi:10.3390/children8070541)
Supplement: Supplementary file 1 [file children-08-00541-s001.zip › children-1242362-supplementary.pdf]

Supplementary material

Table S1. Characteristics and item choices for adults having any children in the household ever experienced risk of choking. Continuous data are reported as median (I, III quartiles); categorical data are reported as a percentage and absolute frequencies. Wilcoxon-Rank sum Tests were performed for continuous variables and the Pearson chi-square test, or Fisher-exact test whatever appropriate, for categorical variables.

Variable	N	no	yes	Combined	P-value
		(N=596)	(N=244)	(N=840)	
**Nation** : Brazil	914	134 (22%)	38 (16%)	172 (20%)	0.024
Italy	-	462 (78%)	206 (84%)	668 (80%)	
**Gender** : *Female*	902	473 (80%)	206 (86%)	679 (82%)	0.055
Male	-	117 (20%)	34 (14%)	151 (18%)	
**Age** of responded (Year)	904	40.0 (34.0-48.0)	42.0 (37.0-48.0)	41.0 (34.0-48.0)	0.112
**Job** : *Housewife*	807	57 (11%)	24 (11%)	81 (11%)	0.747
Unemployed	-	30 (6%)	15 (7%)	45 (6%)	
Teacher/OfficeWorker	-	168 (31%)	65 (31%)	233 (31%)	
Self-employed	-	61 (11%)	26 (12%)	87 (12%)	
Manager	-	117 (22%)	43 (20%)	160 (21%)	
Manual laborer	-	68 (13%)	28 (13%)	96 (13%)	
Retired	-	17 (3%)	6 (3%)	23 (3%)	
Student	-	22 (4%)	3 (1%)	25 (3%)	
**Number of child***: 0*	887	8 (1%)	2 (1%)	10 (1%)	<0.001
1-2	-	50 (9%)	7 (3%)	57 (7%)	
3-4	-	451 (77%)	176 (72%)	627 (75%)	
> 4	-	79 (13%)	59 (24%)	138 (17%)	
**Education** *: highereducation*	895	326 (55%)	123 (50%)	449 (54%)	0.454
primary education	-	73 (12%)	30 (12%)	103 (12%)	
Post-secondaryeducation	-	196 (33%)	91 (37%)	287 (34%)	
**Hazardous items for children < 1 year (First response)**: *Batteries*	894	105 (18%)	38 (16%)	143 (17%)	0.876
Candies	-	88 (15%)	33 (14%)	121 (14%)	
Coins	-	60 (10%)	21 (9%)	81 (10%)	
Hotdog	-	22 (4%)	9 (4%)	31 (4%)	
Jewelry	-	128 (21%)	52 (21%)	180 (21%)	
Nuts	-	7 (1%)	2 (1%)	9 (1%)	
Popcorn	-	55 (9%)	22 (9%)	77 (9%)	
Seeds	-	10 (2%)	8 (3%)	18 (2%)	
Stationery	-	10 (2%)	5 (2%)	15 (2%)	
Toys	-	111 (19%)	54 (22%)	165 (20%)	
**Hazardous items for children < 1 year (Second response)**: *Batteries*	677	14 (3%)	16 (9%)	30 (5%)	0.027
Candies	-	79 (18%)	38 (20%)	117 (18%)	
Coins	-	162 (36%)	54 (29%)	216 (34%)	
Hotdog	-	5 (1%)	6 (3%)	11 (2%)	
Nuts	-	50 (11%)	16 (9%)	66 (10%)	
Popcorn	-	12 (3%)	8 (4%)	20 (3%)	
Seeds	-	58 (13%)	19 (10%)	77 (12%)	
Stationery	-	17 (4%)	9 (5%)	26 (4%)	

Toys	-	50 (11%)	20 (11%)	70 (11%)	
**Hazardous items for children ages 1-2 years (First response)**: *Batteries*	888	109 (18%)	54 (22%)	163 (20%)	0.013
Candies	-	78 (13%)	43 (18%)	121 (15%)	
Coins	-	56 (9%)	19 (8%)	75 (9%)	
Hotdog	-	32 (5%)	19 (8%)	51 (6%)	
Jewelry	-	71 (12%)	21 (9%)	92 (11%)	
Nuts	-	6 (1%)	4 (2%)	10 (1%)	
Popcorn	-	66 (11%)	15 (6%)	81 (10%)	
Seeds	-	3 (1%)	3 (1%)	6 (1%)	
Stationery	-	21 (4%)	17 (7%)	38 (5%)	
Toys	-	150 (25%)	47 (19%)	197 (24%)	
**Hazardous items for children ages 1-2 years (Second response)**: *: Batteries*	677	14 (3%)	5 (3%)	19 (3%)	0.341
Candies	-	137 (31%)	46 (23%)	183 (29%)	
Coins	-	125 (29%)	63 (32%)	188 (30%)	
Hotdog	-	18 (4%)	7 (4%)	25 (4%)	
Nuts	-	57 (13%)	22 (11%)	79 (12%)	
Popcorn	-	8 (2%)	8 (4%)	16 (3%)	
Seeds	-	20 (5%)	9 (5%)	29 (5%)	
Stationery	-	19 (4%)	11 (6%)	30 (5%)	
Toys	-	40 (9%)	25 (13%)	65 (10%)	
**Hazardous items for children ages 3-6 years (First response)**: *Batteries*	886	115 (19%)	56 (23%)	171 (21%)	0.087
Candies	-	86 (15%)	46 (19%)	132 (16%)	
Coins	-	42 (7%)	9 (4%)	51 (6%)	
Hotdog	-	35 (6%)	22 (9%)	57 (7%)	
Jewelry	-	54 (9%)	17 (7%)	71 (9%)	
Nuts	-	6 (1%)	5 (2%)	11 (1%)	
Popcorn	-	60 (10%)	19 (8%)	79 (9%)	
Seeds	-	5 (1%)	4 (2%)	9 (1%)	
Stationery	-	35 (6%)	14 (6%)	49 (6%)	
Toys	-	152 (26%)	50 (21%)	202 (24%)	
**Hazardous items for children ages 3-6 years (Second response)**:: *Batteries*	676	13 (3%)	5 (3%)	18 (3%)	0.237
Candies	-	138 (31%)	45 (23%)	183 (29%)	
Coins	-	130 (30%)	60 (31%)	190 (30%)	
Hotdog	-	19 (4%)	7 (4%)	26 (4%)	
Nuts	-	56 (13%)	22 (11%)	78 (12%)	
Popcorn	-	6 (1%)	7 (4%)	13 (2%)	
Seeds	-	19 (4%)	9 (5%)	28 (4%)	
Stationery	-	20 (5%)	12 (6%)	32 (5%)	
Toys	-	39 (9%)	26 (13%)	65 (10%)	

Table S2. Adults having any children in the household ever experienced risk of choking in managerial position/office and position/teachers category in Italy and Brazil. Data are reported as a percentage and absolute frequencies. Pearson chi-square test or Fisher-exact test has been performed whatever appropriate.

Variable	N	BRASIL	ITALY	Combined	P-value
	-	(N=26)	(N=393)	(N=419)	-
**Have any children in your household ever experienced risk of chocking?**: no	393	22 (85%)	263 (72%)	285 (73%)	0.15
yes	-	4 (15%)	104 (28%)	108 (27%)	-
